# Patient volume and quality of primary care in Ethiopia: findings from the routine health information system and the 2014 Service Provision Assessment survey

**DOI:** 10.1186/s12913-021-06524-y

**Published:** 2021-05-22

**Authors:** Catherine Arsenault, Bereket Yakob, Tizta Tilahun, Tsinuel Girma Nigatu, Girmaye Dinsa, Mirkuzie Woldie, Munir Kassa, Peter Berman, Margaret E. Kruk

**Affiliations:** 1 Department of Global Health and Population, Harvard T.H. Chan School of Public Health, 665 Huntington Avenue, Boston, MA 02115 USA; 2 Fenot Project, Harvard T.H. Chan school of Public Health and School of Population and Public Health, University of British Columbia, Addis Ababa, Ethiopia; 3 Department of Public Health and Health Policy, College of Health Sciences, Haramaya University, Harar, Ethiopia; 4 Ministry of Health, Addis Ababa, Ethiopia; 5 School of Population and Public Health, University of British Columbia, Vancouver, British Columbia Canada

## Abstract

**Background:**

Several studies have reported inadequate levels of quality of care in the Ethiopian health system. Facility characteristics associated with better quality remain unclear. Understanding associations between patient volumes and quality of care could help organize service delivery and potentially improve patient outcomes.

**Methods:**

Using data from the routine health management information system (HMIS) and the 2014 Ethiopian Service Provision Assessment survey + we assessed associations between daily total outpatient volumes and quality of services. Quality of care at the facility level was estimated as the average of five measures of provider knowledge (clinical vignettes on malaria and tuberculosis) and competence (observations of family planning, antenatal care and sick child care consultations). We used linear regression models adjusted for several facility-level confounders and region fixed effects with log-transformed patient volume fitted as a linear spline. We repeated analyses for the association between volume of antenatal care visits and quality.

**Results:**

Our analysis included 424 facilities including 270 health centers, 45 primary hospitals and 109 general hospitals in Ethiopia. Quality was low across all facilities ranging from only 18 to 56% with a mean score of 38%. Outpatient volume varied from less than one patient per day to 581. We found a small but statistically significant association between volume and quality which appeared non-linear, with an inverted U-shape. Among facilities seeing less than 90.6 outpatients per day, quality increased with greater patient volumes. Among facilities seeing 90.6 or more outpatients per day, quality decreased with greater patient volumes. We found a similar association between volume and quality of antenatal care visits.

**Conclusions:**

Health care utilization and quality must be improved throughout the health system in Ethiopia. Our results are suggestive of a potential U-shape association between volume and quality of primary care services. Understanding the links between volume of patients and quality of care may provide insights for organizing service delivery in Ethiopia and similar contexts.

**Supplementary Information:**

The online version contains supplementary material available at 10.1186/s12913-021-06524-y.

## Introduction

As a low-income country with a population of 109 million people and a high fertility rate, Ethiopia is one of the countries with the highest proportion of unmet health care needs [1]. But despite impressive progress in improving health outcomes in the past decade, Ethiopia’s health care system continues to face important challenges. Wide deficits in quality for a range of health services have been documented, revealing poor adherence to evidence-based standards of care [2–6]. Improving the quality of primary care services is crucial to achieving the health-related sustainable development goals. Understanding health system factors and facility characteristics associated with better quality care is a priority.

A positive relationship between higher patient volumes and better quality of care and health outcomes has been shown in prior studies, largely from high-income countries [7–9]. These studies show that patients achieve better outcomes in higher-volume facilities and that similarly, higher-volume surgeons have lower rates of surgical complications and perioperative mortality [10]. Whether volumes also influence the quality of primary care services is less clear. Some studies have shown a positive relationship between patient volume and better-quality care for diabetes and mental health services, while others found that providers with more patients had poorer antibiotic prescribing practices and lower quality care for diabetes [11–14].

There are several potential pathways for patient volumes to influence quality of care. First, higher patient volumes could positively influence quality if providers develop a greater expertise by treating larger numbers of patients and become more skilled. In contrast, larger patient volumes may lead to overcrowding, shorter consultations, provider burnout, and possibly lower quality care. Understanding associations between volume and quality could help in organizing health services and directing patients to the optimal health facilities and provider.

Few studies have investigated the volume-quality association in low-income countries [15]. In addition, because facilities in these countries face vastly different constraints, findings from high-income countries may not be applicable. In the present study, we explore associations between patient volumes and quality of care across the Ethiopian primary health care system. This paper provides an initial empirical exploration of the direction and size of the relationship in this context.

## Methods

Our study is a retrospective analysis of two cross-sectional data sources: the Ethiopian service provision assessment survey plus (ESPA+) and the health management information system (HMIS) [16]. The ESPA+, conducted in 2014, is a cross-sectional assessment of all hospitals in Ethiopia and a representative sample of health centers, health posts and private clinics. Ethiopia’s three-tiered health system includes primary health care units (composed of primary hospitals, health centers and their satellite health posts), general hospitals at the second level, and specialized referral hospitals at the third level. Health posts, staffed exclusively with health extension workers, focus on health promotion and prevention and only provide basic curative care [16]. Health centers, primary hospitals, and general hospitals are expected to offer the full range of basic primary care services in Ethiopia including outpatient services for all age groups, curative care for sick children, child growth monitoring, facility-based child vaccination services, modern methods of family planning, antenatal care, and services for sexually transmitted infections [16]. Referral hospitals, the last level of the system, are primarily focused on advanced specialized care. Our analysis is therefore restricted to health centers, primary hospitals and general hospitals.

The ESPA+ includes different survey modules including service availability and readiness, health provider interviews, observation of consultations and client exit interviews. The health provider interview module included a set of service-specific knowledge questions that measured providers’ knowledge in managing common health conditions including malaria, tuberculosis, postpartum hemorrhage and neonatal asphyxia. Any provider who performed consultations, counselling, or laboratory services and were present in the facility on the day of the visit were eligible. A maximum of fifteen providers were interviewed in each facility. In facilities where fifteen or fewer health care providers were available, all of the providers present on the day of the visit were interviewed. In facilities where more than fifteen providers were available, providers were selected based on whether it was possible to also observe their work. The interviewers attempted to observe a sample of antenatal care, family planning and sick child consultations in each facility that provided these services. The sample of consultations observed was based on the number of clients expected for each service on the day of the survey. Interviewers observed a maximum of 15 consultations for each service per facility and a maximum of five clients per provider. For child health consultations, children younger than 5 years old who presented with an illness rather than an injury or accident were selected for observation. The interviewers observed the consultations and recorded the clinical actions performed and provider-patient interactions using a checklist. The ESPA+, conducted in 2014, is the most recent facility assessment in Ethiopia that contains data from observations of consultations.

We also used data from the HMIS for the same year. The HMIS is a routine data collection system designed to support decision-making and planning in health facilities and various levels of the health system. The Ethiopian HMIS captures over 100 indicators from all health facilities in the country, both public and private [17]. These indicators are largely focused on outputs (i.e. volume of services provided) and on the incidence of various diseases. Indicators are reported directly by health facilities, either monthly, quarterly or yearly.

### Dataset merging

Because the two datasets did not contain common health facility identifiers and the HMIS did not contain reliable geocodes at the time, we created statistical code to link health facilities in the ESPA+ to the HMIS database using region, zone, woreda, and facility names as well as facility types. Merging was also checked manually and validated by local researchers.

### Measures

#### Quality of care

We created a composite measure of quality of care at the facility level based on five indices of competent care and clinical knowledge obtained from the ESPA+. Clinical knowledge scores for malaria and tuberculosis were assessed using clinical vignettes. Malaria and tuberculosis are common health conditions treated in outpatient settings in Ethiopia. The clinical knowledge questions can be found in the ESPA+ instruments [16]. These were developed by the World Bank’s Service Delivery Indicators survey [18]. The clinical knowledge scores were calculated based on the proportion of correct answers and were scaled from 0 to 100.

Competent care was assessed based on provider adherence to standards of care during family planning, antenatal care and sick child care consultations (based on direct observations of consultations). The three scores of adherence to standards of care were built using guidelines from the WHO, where essential elements of care were identified, and matched to those available in the ESPA+ [19–21]. The quality indices were calculated as the percentage of items fulfilled per visit, to provide a continuous quality score scaled from 0 to 100. The individual items included in all three indices are found in supplementary materials. These indices have been used in previous studies [22–24]. To create a facility-level quality of care measure, we took the average score for each of the five measures separately across all consultations observed and all providers interviewed in each facility. Facility-level quality of care was then estimated as the average of the five quality scores.

#### Annual and daily volume of patient visits

Our exposure of interest was the total number of outpatient visits reported in the HMIS for the Ethiopian Financial Year 2007 (12 months ranging from July 2014 to June 2015 according to the Western Calendar), which encompasses the ESPA+ data collection period. Every month, facilities must report total outpatient visits to the HMIS. This requires tallying all visits at the general outpatient clinic, specialty outpatient clinic visits, tuberculosis clinics, antiretroviral clinics, voluntary counseling and testing clinics, maternal and child health clinics (vaccination, well baby clinics, antenatal care, postnatal care, family planning etc.), emergency department, and dressing and injection room visits. The outpatient volume is reported as a total.

We estimated daily outpatient volume by dividing the annual volume by the number of days per year during which primary care services were offered in each facility (as reported in the ESPA+). We also calculated the average number of antenatal care consultations provided per day, by dividing the annual antenatal care volume by the number of days during which antenatal care services were offered at the facility.

Because data from the HMIS are self-reported and may contain errors, we excluded any facilities with inconsistent reports. For example, we excluded any facility where the number of outpatient visits reported for the year was lower than the sum of antenatal care and postnatal care visits or family planning consultations which are reported individually in the HMIS. In addition, despite being active, a few facilities reported no outpatient visits for the year and were excluded.

#### Covariates

We identified a series of covariates that could potentially influence the quality of care provided. These included facility location (urban vs. rural), facility type (health center, primary hospital or general hospital) and the total number of clinical staff providing primary care services (medical doctors, health officers and nurses) and structural quality. Structural quality was assessed using the World Health Organization’s service availability and readiness assessment guidelines [25]. We assessed availability of basic equipment using a binary indicator for having at least one functional of each of the following: scale, pediatric scale, thermometer, stethoscope, BP apparatus and exam light. We also included an essential medicine index calculated as the average of 19 medications available at the facility (list in supplementary materials). The medicine index was a continuous score ranging from 0 to 1.

#### Statistical analysis

Because the volume of outpatient visits was highly skewed (supplementary materials) we transformed the variable by taking its natural log. To visualize the unadjusted association, we plotted log-transformed outpatient volume and facility-level quality and fitted a locally weighted smoothing (loess) curve. To approximate the non-linear relationship between the two variables, we fitted the log-transformed volume as a linear spline and used a linear regression model to assess its association with quality.

We tested several models with one, two and three knots at different locations and selected the best model fit according to the Akaike’s and Schwarz’s Bayesian information criteria (AIC and BIC) [26, 27]. The different models tested are described in supplementary materials. The knots were selected independent of the outcome results. The final regression model included 11 region fixed effects and was adjusted for several confounders at the facility-level. To aid in interpreting the results, the coefficients for the volume variable were transformed to express the estimated difference in quality for a doubling in volume.

We performed three sensitivity analyses. First, we repeated the analysis using sampling weights obtained from the ESPA+. Second, we looked at the volume-quality association within strata of facility types (health centers vs. hospitals). Third, we assessed associations between volume and quality for antenatal care specifically. We modelled the association between volume of antenatal care visits for the year (as provided by the HMIS) and antenatal care quality (estimated using observations of antenatal care consultations and based on providers adherence to standards of care) [20]. All analyses were performed using STATA version 15.

## Results

The ESPA+ surveyed a total of 1165 facilities, including 474 general hospitals, primary hospitals and health centers. Of those 474 facilities, 450 were successfully linked to their HMIS records. However, 15 facilities reported no outpatients visits for the year and were excluded. Another 11 were excluded because the total outpatient visits were lower than the sum of antenatal care, postnatal care or family planning consultations. This led to a final analytical sample of 424 facilities including 109 general hospitals, 45 primary hospitals and 270 health centers. This included 44 private facilities and 380 government-owned facilities.

Using data from observations and clinical vignettes, we estimated an overall quality score for each facility. Across the 424 facilities included, 3350 providers completed the clinical vignettes and 3629 primary care consultations were observed by the ESPA+ (average number of observations and providers interviewed per facility can we found in supplementary materials. We found low quality of care across all facilities with scores ranging from only 18 to 56% and a mean of 38% (distribution in supplementary materials). Across the five quality indices, scores were lowest for the malaria vignette (Table 1). Average score on the tuberculosis vignette was 41%. Average adherence to standards of care was approximately 35% for both family planning and sick child care consultations and 49% for antenatal care.

**Table 1:** Characteristics of facilities included, health management information system and the Ethiopian service provision assessment survey plus, 2014

	General hospitals	Primary hospitals	Health centers	Total
	(***N*** = 109)	(***N*** = 45)	(***N*** = 270)	(***N*** = 424)
	Mean (SD)	Mean (SD)	Mean (SD)	Mean (SD)
**Quality of care** ^a^				
Adherence to standards of care:				
Family planning	34.65 (10.58)	32.76 (10.45)	36.30 (11.00)	35.45 (10.85)
Antenatal care	49.07 (10.84)	48.71 (7.46)	48.20 (11.40)	48.54 (10.78)
Sick child care	33.10 (10.20)	36.84 (11.08)	36.10 (12.88)	35.31 (11.99)
Clinical knowledge:				
Malaria	32.58 (7.98)	36.13 (8.31)	31.03 (8.92)	31.97 (8.75)
Tuberculosis	42.96 (9.06)	43.06 (7.54)	40.32 (9.20)	41.29 (9.07)
Overall quality score	38.76 (6.09)	39.61 (6.16)	37.01 (6.76)	37.73 (6.59)
**Facility characteristics**				
Outpatient volume (patients/day)	116.34 (111.37)	91.53 (58.41)	29.78 (34.26)	58.58 (75.98)
Number of primary care staff ^b^	65.92 (36.19)	32.91 (19.60)	11.24 (8.93)	27.59 (31.26)
Essential medicine index ^c^	0.72 (0.13)	0.68 (0.13)	0.44 (0.15)	0.54 (0.19)
	**N (%)**	**N (%)**	**N (%)**	**N (%)**
Basic equipment ^d^	82 (75.2%)	35 (77.8%)	120 (44.4%)	237 (55.9%)
Urban location	98 (89.9%)	40 (88.9%)	87 (32.2%)	225 (53.1%)

^a^ Family planning, antenatal care and sick child care consultations were observed in 235, 282 and 338 facilities, respectively. Clinical knowledge scores were available in all 424 facilities

^b^ Includes medical doctors, health officers and nurses

^c^ Average of 19 essential medicines listed in supplementary materials

^d^ Binary indicator for the availability of at least one functional: scale, pediatric scale, thermometer, stethoscope, BP apparatus and exam light

Outpatient volumes differed considerably across facility types ranging from less than one patient seen at the facility per day to 581. On average, health centers had 30 outpatient visits per day, primary hospitals had 92, and general hospitals reported 116 outpatient visits per day. Number of staff available for primary care services (nurses, health officers and medical doctors) and structural quality were also higher in hospitals compared to health centers (Table 1). The number of primary care providers ranged from only 1 to 188 per facility, with nurses representing the majority. Most health centers had no medical doctors, with nurses and health officers providing most of the care. Most hospitals were in urban areas, while 68% of health centers were in rural areas. Only 56% of facilities had all six basic equipment items recommended for general outpatient departments by the WHO’s service availability and readiness assessment.

**Fig. 1:**
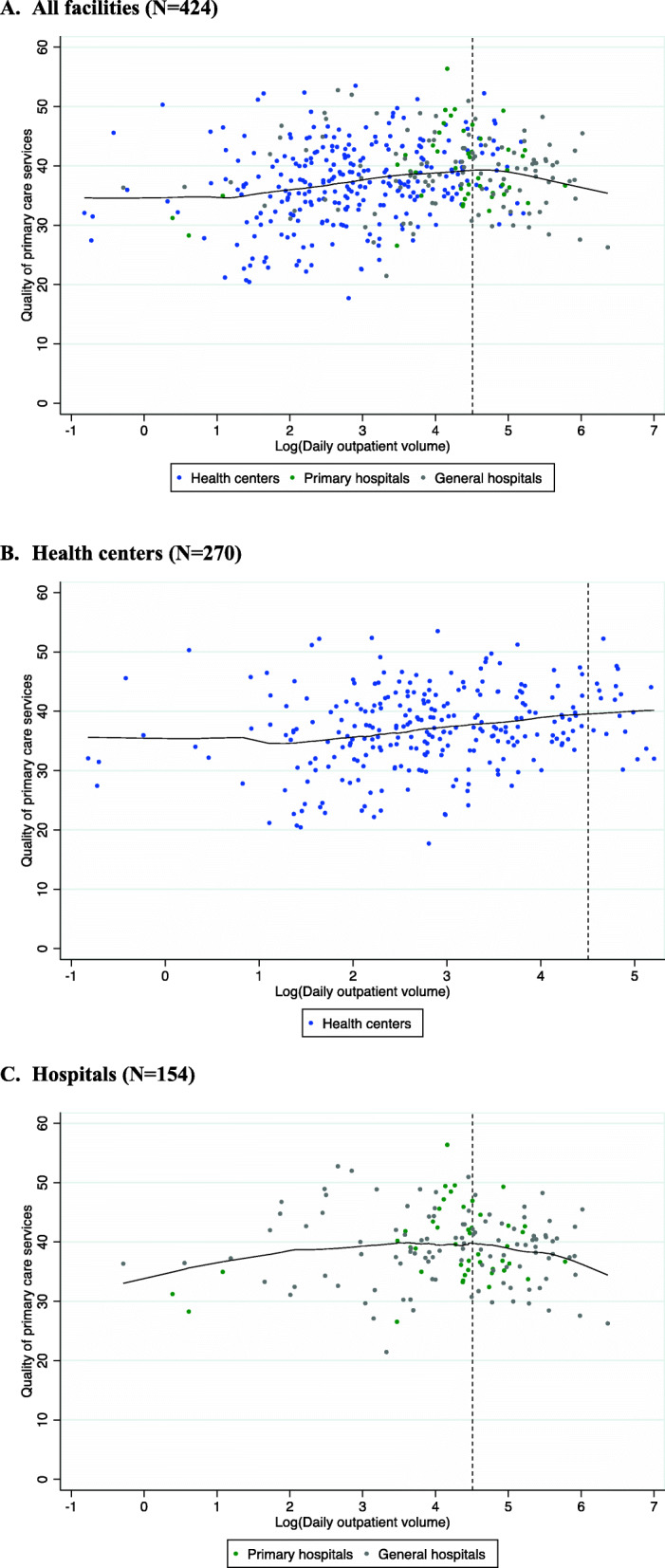
Association between log-transformed outpatient volume and quality of care at the facility level in Ethiopia, 2014. **a** All facilities (*N* = 424). **b** Health centers (*N* = 270). **c** Hospitals (*N* = 154). Black line is the locally weighted smoothing (loess) curve. Dotted line represents the linear spline at 90.6 outpatients per day (4.5 on the log scale)

In the regression analysis, outpatient volume was fit as a linear spline with one knot at 90.6 outpatient visits per day. This was equivalent to the 80th percentile of outpatient volume. This knot was chosen because the resulting model had the lowest AIC and BIC across 10 models tested with one, two or three knots at different locations. Figure 1 shows unadjusted associations between outpatient volume and quality of care in all facilities (A), health centers (B) and hospitals (C).

In the multivariable regression model (combining all facilities) we found a small, but statistically significant, association between outpatient volume and quality of care which appeared non-linear, with an inverted U-shape (Table 2). Among facilities with less than one to 90.6 outpatient visits per day, a doubling in volume was associated with a 0.78 of a percentage-point increase in quality (95% CI 0.24, 1.32) (Table 2). However, among facilities with more than 90.6 outpatient visits per day, a doubling in volume was associated with a 2.34 percentage-points decrease in quality (95% CI -4.18, − 0.50). Having all six basic equipment items was also associated with higher quality of care. Analyses using sampling weights yielded similar results.

**Table 2:** Results of a linear regression model of quality of care at the facility level in Ethiopia, 2014, *N* = 424

	Coefficient ^**a**^	95% CI	***p***-value
**Facility characteristics**			
Daily outpatient volume ^b^			
Less than 90.6	0.78	(0.24, 1.32)	0.005**
90.6 +	− 2.34	(−4.18, − 0.50)	0.013*
Number of primary care staff ^c^	−0.01	(− 0.04, 0.03)	0.702
Basic equipment ^d^	2.01	(0.68, 3.35)	0.003**
Essential medicine index ^e^	4.36	(−0.69, 9.41)	0.090
Urban location	−0.98	(−2.63, 0.66)	0.241
Facility type			
General hospital	ref.		
Primary hospital	−0.16	(−2.65, 2.34)	0.901
Health center	−0.65	(−3.02, 1.73)	0.592
R-squared	0.1518		

^a^ Model includes 11 region fixed effects (not shown).

^b^ Linear spline with one knot at 90.6 outpatients per day. The estimated difference represents a 100% increase (doubling) in volume.

^c^ Includes medical doctors, health officers and nurses.

^d^ Binary indicator for the availability of at least one functional among: scale, pediatric scale, thermometer, stethoscope, BP apparatus and exam light.

^e^ Average of 19 essential medicines listed in supplementary materials.

* *p*-value ≤0.05 ** *p*- value ≤0.01

Results stratified by facility type are shown in supplementary materials and were consistent with those from the main analysis. In health centers, the positive volume-quality association in lower-volume facilities remained significant whereas in hospitals, the negative association between volume and quality remained significant in facilities seeing more than 90.6 patients per day.

We repeated the analysis looking at the association between volume of antenatal care consultations and antenatal care quality. Number of antenatal care consultations ranged from less than one to 50.5 per day per facility, with a mean of 5.4 antenatal care consultations per day. Quality of antenatal care was low, on average providers performed only 48.54% or recommended care (Table 1). The regression analysis for the association between antenatal care volume and quality is shown in supplementary materials. We fit log-transformed antenatal care volume as a linear spline with one knot at 11.5 visits per day. This was equivalent to the 90th percentile of log-transformed daily volume, and this model was chosen because it had the lowest AIC and BIC across 10 models tested (supplementary materials). We found results similar to those for outpatient visits. In facilities seeing between less than one and 11.5 antenatal care clients per day, a doubling in volume was associated with a 1.5 percentage-points increase in antenatal care quality (95% CI 0.3, 2.6). However, in facilities seeing more than 11.5 women per day, a doubling in volume was associated with a 7.0 percentage-points decrease in antenatal care quality (95% CI -13.2, − 0.8).

## Discussion

Using data from a facility survey and the HMIS in 424 facilities in Ethiopia, we found a small association between patient volume and quality of primary care. The association appeared non-linear with an inverted U-shape. Patient volumes were positively associated with quality until a certain threshold, after which quality may be decreasing with higher patient volumes. Quality of care was extremely low overall, ranging from only 18 to 56% across facilities with a mean of 38%. Thus, the magnitude of association between volume and quality was also small (a doubling in volume would be associated with a 0.78 percentage point increase in quality only). Interpretation of these results should therefore be done with caution. These analyses were conducted to explore associations rather than imply causation. Future research should repeat this analysis in a different context and using more recent data, with ideally more variation in quality. Nonetheless, our results are suggestive of a U-shape association between volume and quality.

Implications of these findings are threefold. First and foremost, our study showed that the quality of primary care services in Ethiopia is poor across all facilities. Patients seeking family planning, antenatal care or care for their sick child received less than half of recommended clinical actions on average during consultations. Providers also showed poor knowledge and diagnostic accuracy for malaria and tuberculosis. Facilities were also poorly staffed and many lacked basic equipment and essential medicine. However, it is important to acknowledge that these data are from 2014 and the situation may have changed since. Nonetheless, no other national health facility survey with observations of care has been conducted since.

Second, a potential positive volume-quality relationship in lower-volume facilities could indicate that seeing more patients may improve provider knowledge and practice. In lower-volume facilities, providers may only see a few cases of each type every month which may not be sufficient to maintain the skills needed to provide each service adequately. In larger facilities, the same service may be provided to a larger number of individuals every day, allowing providers to develop a greater expertise with a particular service, while also retaining a manageable world load. Providers in lower-volume facilities may therefore require more support to maintain skills. Nonetheless, given the widespread poor-quality care in Ethiopia, overall strategies to improve quality should target all facilities.

Third, the small negative association between volume and quality in higher-volume facilities may indicate that large facilities are not be the optimal location to provide primary care. Providers in large facilities such as hospitals may see too many patients and be too rushed to complete all of the recommended clinical tasks for non-urgent services such as family planning, antenatal care or non-severe illnesses. Several studies, largely from high-income countries, have found that doctors with longer consultations tend to achieve better outcomes and higher levels of patient satisfaction [28]. Nonetheless, it is estimated that primary care providers spend less than 5 mins per patient on average in low and middle-income countries [29]. Future research should investigate the length and quality of primary care consultations in higher-volume facilities.

These findings have implications for the design of health systems. Higher-volume facilities with advanced diagnostic and treatment capacities in Ethiopia (such as general hospitals) are currently providing basic primary care services to stable, non-urgent patients. It is estimated that around 20% of all outpatient consultations in Ethiopia take place in hospitals rather than health centers, health posts or clinics [30]. A study across 56 LMICs also found that between 17 and 23% of people use hospitals for primary care, rather than health centers or clinics [31]. Meanwhile, many hospitals in LMICs struggle to provide high-quality specialized and surgical care and to save the lives of those with complex injuries or obstetric complications [32, 33]. In turn, rather than focusing solely on basic primary care, health centers in Ethiopia are expected to handle more complex care such as childbirth. Clearly defining the functions and capacities of different levels of care and allowing providers to focus on the tasks that they do best, could be an effective way to improve quality in Ethiopia. Keeping equity of access in mind, policy makers should consider the feasibility of redirecting primary care services from hospitals to health centers and clinics capable of providing quality care.

Our study also highlights the importance and feasibility of using routine HMIS data for health system research. Concerns about data quality have hampered the use of HMIS data in health services research. Nonetheless, verification studies in Ethiopia have shown good level of agreement between the volume of services reported monthly in the HMIS and totals found in source documents (e.g., paper-based registers used in facilities). For example, the EPHI found that the number of antenatal care visits reported in the HMIS were within 8% on average of totals found in antenatal care registries while the number of deliveries were within 1% [34]. Endriyas and colleagues found that service volumes were between 1 and 7% of source documents on average in the Southern Nations, Nationalities, and Peoples’ Region (SNNPR) of Ethiopia [35]. Work remains to improve the accuracy of these data in Ethiopia and elsewhere. Ethiopia has recently adopted the DHIS2 platform along with 73 other low- and middle-income countries worldwide. A recent review found that only 41 articles had been published since 2011 using DHIS2 data [36]. Greater utilization of these data by researchers and policy makers, combined with feedback, would contribute toward further improving data quality.

Several prior studies have described poor levels of quality of care in Ethiopia and have investigated potential determinants of quality [2–4, 6, 37–40]. Studies have shown deficiencies in structural quality (lack of equipment, medicine, supplies and human resources) and provider knowledge, lack of adherence to standards of care, poor content of care, and moderate levels of patient satisfaction. The facility characteristics associated with better quality care varied across these studies and remain unclear in Ethiopia. To our knowledge, our study is the first to investigate associations between volume of patients and quality of care in the Ethiopian context. Though we found a very small association between volume and quality, our conclusions were robust to stratification by facility type, and we found a similar shape of association when assessing the relationship between volume and quality for antenatal care visits specifically.

### Limitations

Nonetheless, our study has several limitations. First, HMIS data is self-reported by health facilities and may contain errors. We excluded 26 facilities with inconsistent data. Many of these were private and may have had fewer incentives to correctly report to the HMIS. Of the 55 private facilities included in the initial sample, 11 (20%) had to be excluded due to poor HMIS reporting, while only 15 of the 395 (4%) public facilities were excluded due to poor reporting. It is unclear how misreporting of the patient volumes may have biased our analysis. Higher quality facilities may be more likely to correctly report to the HMIS. However, whether poorer quality facilities tend to over- or under-report patient volumes is unclear. Second, our measure of primary care quality is limited and does not consider other dimensions of quality such as user experience or patient outcomes. It is also based on observations of consultations on the day of the survey and on vignettes completed by health care workers who were present that day. It may therefore not reflect the full scope of provider competence and knowledge in each facility. Nonetheless, direct observations of care and clinical vignettes are recognized measures of quality that reflect the quality of the processes of care available to patients in these facilities. Third, the data are several years old (collected/reported in 2014). Few measures of health care quality exist in Ethiopia and national facility surveys with direct observations of care have not been conducted since. Fourth, health posts were not included in this analysis. There were few consultations observed and no clinical vignettes conducted in health posts by the ESPA+. Fifth, this was a facility-level analysis and it was not possible to determine provider-specific volumes. Inferences on the association between patient volumes and provider behaviors should be interpreted with cautions. Future research should investigate associations between provider-specific patient volumes and quality of care in low-income countries. Finally, we were unable to obtain reliable data on facility catchment population which could confound the association between volume and quality. In addition, it is worth noting that the association between quality and volume could be bi-directional as higher-quality facilities may attract more patients.

## Conclusions

Quality of primary care services (including provider knowledge and practice) must be improved to achieve better health outcomes and improve people’s confidence in the health system in Ethiopia. The identification of a potential association between volume and quality of primary care has implications for future research and for the organization of health services. Improving the quality of primary care services may involve reorganizing service delivery and potentially shifting the different services provided at different levels of the health system.

## Supplementary Information

**Additional file 1.**

## Data Availability

The data that support the findings of this study were obtained from the Ethiopian Ministry of Health and the Ethiopian Public Health Institute, but restrictions apply to the availability of these data, which were used under license for the current study, and are not publicly available. Data are however available from the authors upon reasonable request and with permission of the Ethiopian Public Health Institute and Ethiopian Ministry of Health.
