# Supplementary material for: Patient volume and quality of primary care in Ethiopia: findings from the routine health information system and the 2014 Service Provision Assessment survey
Source: BMC Health Serv Res. 2021 May 22;21:485. doi: 10.1186/s12913-021-06524-y (PMC8140434; doi:10.1186/s12913-021-06524-y)
Supplement: Supplementary file 1 — Additional file 1. [file 12913_2021_6524_MOESM1_ESM.docx]

**Supplementary materials**

**Patient volume and quality of primary care in Ethiopia: findings from the routine health information system and the 2014 Service Provision Assessment survey**

**Table 1.** Items included in the adherence to standards of care scores

| **Service** | **Index components: Recommended care items** |
| --- | --- |
| Family planning | Asked: age, current breastfeeding, # living children, chronic illness history, reproductive intentions, last delivery, last period, menstruation regularity, smoking history, STI symptoms, desired timing  Exam: blood pressure, pelvic exam, weight  Prescribed at least one family planning method  Counseling: explained how to use method, possible side effects and what to do if have problems, discussed partner status and attitude, risk of STI/HIV, condoms, dual method use, asked about concerns, discussed follow-up visit  Privacy: ensured visual and auditory privacy, assured client confidentiality  Communication: Used visual aids, checked and wrote on card |
| Antenatal care | Asked: danger signs, pregnancy history, last period, previous complications^a^  Exam: weight, fundal height, oedema, vaginal exam, blood pressure, ultrasound, fetal heart rate^a^  Test: syphilis, HIV, anemia, blood group, urine  Prevention: tetanus toxoid injection, iron/folic acid, IPTp against malaria^a^  Counseling: nutrition, sleeping under ITN, birth plan, supplies for home delivery, breastfeeding^a^, postpartum/postnatal care^a^, pregnancy spacing^a^, wrote on card |
| Sick child care | Asked: ability to drink, normal and sick feeding pattern^b^, cough or difficulty breathing, diarrhea, fever, vomiting, convulsions, maternal HIV status, ear problems  Exam: weight, plots weight on chart, temperature, pallor, oedema, MUAC, jaundice, count respirations, check mouth^b^  Gives or checks card for: immunizations, vitamin A, deworming  Counseling: how to administer meds if prescribed, gives directions for feeding, danger signs, scheduled or discussed return visit, gave diagnosis, keeping infant warm^b^ |

^a^ Follow-up antenatal care visits only

^b^ For children under 2 months old only

**Table 2.** Items included in the clinical knowledge scores

| **Service** | **Questions** | **Expected answers** |
| --- | --- | --- |
| **Tuberculosis care** | 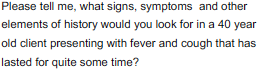 | Duration of cough, type of cough (productive, dry, etc.), presence of blood in sputum, chest pain/difficulty breathing, presence of fever and pattern, pattern of sweats (night), history of TB in household, history of contact with an adult with prolonged cough in the household, HIV test taken/HIV sero-status, weight loss, appetite, general health condition (tiredness/fatigue), others in household with same type of cough, has this happened before, medication/treatment history, drinking/alcohol consumption, smoking history, normal diet, profession, high risk sexual behavior. |
|  | 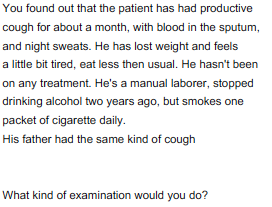 | Take temperature, check weight, check height, take pulse rate, take respiratory rate, chest examination (auscultation or other), retraction/decrease movement, take blood pressure, refer to other provider or facility. |
|  | 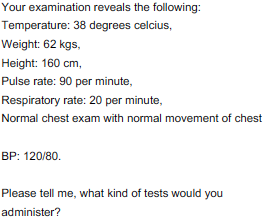 | Sputum for AFB (taken spot), Chest X-Ray, ESR (Erythrocytic sedimentation rate), hemogram/full blood count, hemoglobin, HIV test, blood sugar test. |
|  | 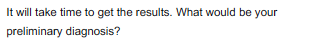 | Pulmonary TB |
|  | 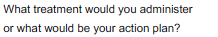 | 4 drugs for 2 months and 2 drugs for 6 months OR follow-up in the TB clinic or refer to another provider or facility.  2 drugs can be: rimactazid and rifampicin, ethambutol & pyrazinamide, and ethambutol and isoniazid. |
|  | 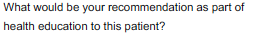 | Adherence to treatment and contact testing. |
| **Malaria care** | 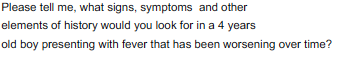 | Duration of fever, presence of fever and pattern, history of convulsions, history of vomiting, loss of appetite/changes in eating habits, diarrhea, cough or difficulty breathing, vaccination status to date. |
|  | 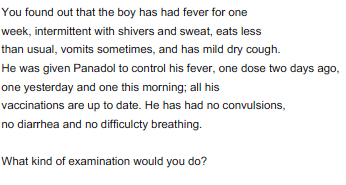 | Take temperature, take weight, check hands for palmar pallor, take respiratory rate, check for swollen feet, check responsiveness, check for neck stiffness, check skin condition check tongue. |
|  | 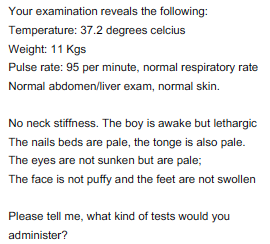 | Microscopy/blood slide for malaria parasite (BF) or rapid diagnostic test, hemoglobin or full blood count |
|  | 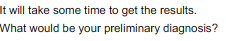 | Malaria with anemia |
|  | 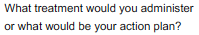 | Artemether/Lumefantrine 6 dose regiment, 2 tablets per dose, adequate fluid and nutrition, or chloroquine, quinine intravenous infusion or rectal artesunate (100 mg suppository). |
|  | 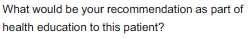 | Adherence to treatment, prompt return if symptoms worsen. |

**Table 3.** Availability of essential medicines

| **Average of 19 medications available at the facility:** |
| --- |
| Amitriptyline tablet, Amlodipine tablet or alternative, Amoxicillin syrup/suspension or dispersible tablet, Amoxicillin tablet, Ampicillin powder, Beclomethasone inhaler, Ceftriaxone injection, Enalapril tablet or alternative ACE inhibitor, Gentamicin injection, Glibenclamide tablet, Ibuprofen / brufen tablet, Insulin injection, Metformin tablet, Omeprazole tablet or alternative, Oral rehydration solution, Paracetamol tablet, Salbutamol inhaler, Simvastatin tablet or other statin, Zinc sulphate tablet or syrup |

**Table 4.** Average and total number of clinical observations and provider interviewers (clinical vignettes) performed per facility type

|  | **General hospitals** | | **Primary hospitals** | | **Health centers** | | **Total** | |
| --- | --- | --- | --- | --- | --- | --- | --- | --- |
|  | **(N = 109)** | | **(N = 45)** | | **(N = 270)** | | **(N = 424)** | |
|  | **Total** | **Mean** | **Total** | **Mean** | **Total** | **Mean** | **Total** | **Mean** |
| Malaria vignettes completed | 1,164 | 10.7 | 449 | 10.0 | 1,737 | 6.4 | 3,350 | 7.9 |
| Tuberculosis vignettes completed | 1,164 | 10.7 | 449 | 10.0 | 1,737 | 6.4 | 3,350 | 7.9 |
| Observations of family planning consultations | 241 | 2.4 | 119 | 2.9 | 514 | 2.6 | 874 | 2.6 |
| Observations of antenatal care consultations | 559 | 5.6 | 193 | 4.7 | 614 | 3.1 | 1,366 | 4.0 |
| Observations of sick child care consultations | 503 | 5.1 | 207 | 5.1 | 679 | 3.4 | 1,389 | 4.1 |
|  |  |  |  |  |  |  |  |  |

**Figure 1.** Volume of outpatient visits* per day from July-June 2014 in Ethiopia, N= 424 facilities, Source: Health Management Information System (HMIS)

* before log-transformation

**Table 5.** Location and number of knots and model selection criteria for the association between log-transformed daily outpatient volume and quality of care

| **Number of knots** | **Knot location expressed in percentiles of volume** | | | **AIC** | **BIC** |
| --- | --- | --- | --- | --- | --- |
| **3** | 0.10 | 0.50 | 0.90 | 2775.677 | 2864.771 |
| **3** | 0.05 | 0.25 | 0.75 | 2770.865 | 2855.909 |
| **3** | 0.25 | 0.50 | 0.75 | 2772.46 | 2857.504 |
| **3** | 0.05 | 0.50 | 0.90 | 2776.178 | 2857.172 |
| **2** |  | 0.50 | 0.90 | 2773.347 | 2854.342 |
| **2** |  | 0.25 | 0.75 | 2771.607 | 2852.602 |
| **2** |  | 0.10 | 0.75 | 2770.035 | 2851.029 |
| **2** |  | 0.05 | 0.75 | 2769.782 | 2850.777 |
| **2** |  | 0.05 | 0.80 | 2769.23 | 2850.225 |
| **1** |  |  | 0.80 | **2769.081** | **2846.026** |

AIC and BIC are Akaike's and Schwarz's Bayesian information criteria.

Percentile of the log-transformed ambulatory volume

variable | p5 p10 p25 p50 p75 p90

-------------+------------------------------------------------------------

lnday | 1.275702 1.601461 2.358232 3.373981 4.373824 4.999853

--------------------------------------------------------------------------

**Figure 2.** Facility-level quality of care* in 424 facilities, Ethiopian Service Provision Assessment Survey, 2014 (average of five scores at the facility level)

Based on adherence to standards of care during direct observations of family planning, antenatal care and sick child care consultations and provider vignettes for malaria and tuberculosis. Average across the five scores at the facility.

**Table 6.** Results of a linear regression model of quality of care at the facility level in Ethiopia, 2014, health centers only (N=270)

|  | **Coefficient ^a^** | **95% CI** | **p-value** |
| --- | --- | --- | --- |
| **Facility characteristics** |  |  |  |
| Daily outpatient volume ^b^ |  |  |  |
| Less than 90.6 | 0.94 | (0.18, 1.70) | 0.015* |
| 90.6 + | 0.27 | (-6.26, 6.80) | 0.936 |
| Number of primary care staff ^c^ | -0.001 | (-0.14, 0.14) | 0.996 |
| Basic equipment ^d^ | 2.76 | (1.10, 4.41) | 0.001** |
| Essential medicine index ^e^ | 1.97 | (-4.85, 8.78) | 0.570 |
| Urban location | -1.44 | (-3.51, 0.62) | 0.170 |
| R-squared | 0.1541 |  |  |

a Model includes 11 region fixed effects.

b Linear spline with one knot at 90.6 outpatients per day. The estimated difference represents a 100% increase (doubling) in volume.

c Includes medical doctors, health officers and nurses.

d Binary indicator for the availability of at least one functional: scale, pediatric scale, thermometer, stethoscope, BP apparatus and exam light.

e Average of 19 essential medicines listed in supplementary materials.

* p-value ≤ 0.05 ** p- value ≤ 0.01 *** p- value ≤ 0.001

**Table 7.** Results of a linear regression model of quality of care at the facility level in Ethiopia, 2014, hospitals only (N=154)

|  | **Coefficient ^a^** | **95% CI** | **p-value** |
| --- | --- | --- | --- |
| **Facility characteristics** |  |  |  |
| Daily outpatient volume ^b^ |  |  |  |
| Less than 90.6 | -0.03 | (-0.96, 0.90) | 0.945 |
| 90.6 + | -2.25 | (-4.18, -0.32) | 0.023* |
| Number of primary care staff ^c^ | 0.001 | (-0.04, 0.04) | 0.949 |
| Basic equipment ^d^ | 0.62 | (-1.68, 2.92) | 0.593 |
| Essential medicine index ^e^ | 6.91 | (-1.36, 15.19) | 0.101 |
| Urban location | 1.91 | (-1.65, 5.46) | 0.291 |
| R-squared | 0.2250 |  |  |

a Model includes 11 region fixed effects.

b Linear spline with one knot at 90.6 outpatients per day. The estimated difference represents a 100% increase (doubling) in volume.

c Includes medical doctors, health officers and nurses.

d Binary indicator for the availability of at least one functional: scale, pediatric scale, thermometer, stethoscope, BP apparatus and exam light.

e Average of 19 essential medicines listed in supplementary materials.

* p-value ≤ 0.05 ** p- value ≤ 0.01 *** p- value ≤ 0.001

**Figure 3.** Number of antenatal care consultations* per day from July-June 2014 in Ethiopia, N= 291 facilities, Source: Health Management Information System (HMIS)

**Table 8.** Location and number of knots and model selection criteria for the associations between log-transformed daily antenatal care volume and antenatal care quality

| **Number of knots** | **Knot location expressed in percentiles of volume** | | | **AIC** | **BIC** |
| --- | --- | --- | --- | --- | --- |
| **3** | 0.10 | 0.50 | 0.90 | 2216.168 | 2293.308 |
| **3** | 0.05 | 0.25 | 0.75 | 2220.378 | 2297.518 |
| **3** | 0.25 | 0.50 | 0.75 | 2218.982 | 2296.122 |
| **3** | 0.05 | 0.50 | 0.90 | 2220.917 | 2294.383 |
| **2** |  | 0.50 | 0.90 | 2215.812 | 2289.279 |
| **2** |  | 0.25 | 0.75 | 2219.166 | 2292.633 |
| **2** |  | 0.10 | 0.75 | 2219.021 | 2292.487 |
| **2** |  | 0.05 | 0.75 | 2219.11 | 2292.576 |
| **2** |  | 0.05 | 0.80 | 2219.429 | 2292.895 |
| **1** |  |  | 0.90 | **2214.297** | **2284.091** |

AIC and BIC are Akaike's and Schwarz's Bayesian information criteria.

Percentile of the log-transformed antenatal care volume

variable | p5 p10 p25 p50 p75 p90

-------------+------------------------------------------------------------

lnANC | -.2177763 .2915883 .5874605 1.343973 1.914704 2.440439

--------------------------------------------------------------------------

**Table 9.** Results of a linear regression model of antenatal care quality at the facility level in Ethiopia, 2014, N=291

|  |
| --- |
| \|  \| **Coefficient ^a^** \| **95% CI** \| **p-value** \| \| --- \| --- \| --- \| --- \| \| **Facility characteristics** \|  \|  \|  \| \| Daily number of antenatal care visits ^b^ \|  \|  \|  \| \| Less than 11.5 \| 1.47 \| (0.31, 2.63) \| 0.013* \| \| 11.5 + \| -7.00 \| (-13.21, -0.79) \| 0.027* \| \| Number of primary care staff ^c^ \| 0.02 \| (-0.04, 0.09) \| 0.420 \| \| Basic equipment ^d^ \| 3.42 \| (0.64, 6.19) \| 0.016* \| \| Essential medicine index ^e^ \| 3.94 \| (-6.40, 14.27) \| 0.454 \| \| Urban location \| -0.82 \| (-4.10, 2.47) \| 0.625 \| \| Facility type \|  \|  \|  \| \| General hospital \| ref. \|  \|  \| \| Primary hospital \| 0.23 \| (-4.43, 4.89) \| 0.922 \| \| Health center \| 3.21 \| (-1.27, 7.69) \| 0.159 \| \| R-squared \| 0.1261 \|  \|  \| |

a Model includes 11 region fixed effects.

b Linear spline with one knot at 11.5 antenatal care visits per day. The estimated difference represents a 100% increase (doubling) in number of ANC visits.

c Includes medical doctors, health officers and nurses.

d Binary indicator for the availability of at least one functional: scale, pediatric scale, thermometer, stethoscope, BP apparatus and exam light.

e Average of 19 essential medicines listed in supplementary materials.

* p-value ≤ 0.05 ** p- value ≤ 0.01 *** p- value ≤ 0.001

**Figure 4.** Association between log-transformed volume of daily antenatal care consultations and antenatal care quality in Ethiopia, 2014

Black line is the locally weighted smoothing (loess) curve. Dotted line represents the linear spline at 11.5 ANC consultations per day.
